# Case Report: *DNAAF4* Variants Cause Primary Ciliary Dyskinesia and Infertility in Two Han Chinese Families

**DOI:** 10.3389/fgene.2022.934920

**Published:** 2022-07-12

**Authors:** Ting Guo, Chenyang Lu, Danhui Yang, Cheng Lei, Ying Liu, Yingjie Xu, Binyi Yang, Rongchun Wang, Hong Luo

**Affiliations:** ^1^ Department of Pulmonary and Critical Care Medicine, The Second Xiangya Hospital, Central South University, Changsha, China; ^2^ Research Unit of Respiratory Disease, Central South University, Changsha, China; ^3^ Hunan Diagnosis and Treatment Center of Respiratory Disease, Changsha, China

**Keywords:** primary ciliary dyskinesia, scoliosis, *DNAAF4*, bronchiectasis, situs inversus, asthenoteratozoospermia

## Abstract

**Background** **:** Primary ciliary dyskinesia (PCD) is a rare genetic disorder, predominantly autosomal recessive. The dynein axonemal assembly factor 4 (*DNAAF4*) is mainly involved in the preassembly of multisubunit dynein protein, which is fundamental to the proper functioning of cilia and flagella. There are few reports of PCD-related pathogenic variants of *DNAAF4*, and almost no *DNAAF4*-related articles focused on sperm phenotype. Moreover, the association between *DNAAF4* and scoliosis has never been reported, to the best of our knowledge.

**Materials and Methods:** We recruited two patients with a clinical diagnosis of PCD. One came from a consanguineous and another from a non-consanguineous family. Clinical data, laboratory test results, and imaging data were analyzed. Through whole exome sequencing, immunofluorescence, electron microscopy, high-speed video microscopy analysis, and hematoxylin–eosin (HE) staining, we identified the disease-associated variants and validated the pathogenicity.

**Results:** Proband 1 (P1, F1: II-1), a 19-year-old man, comes from a non-consanguineous family-I, and proband 2 (P2, F2: II-1), a 37-year-old woman, comes from a consanguineous family-II. Both had sinusitis, bronchiectasis, situs inversus, and scoliosis. P1 also had asthenoteratozoospermia, and P2 had an immature uterus. Two homozygous pathogenic variants in *DNAAF4* (NM_130810.4), c.988C > T, p.(Arg330Trp), and *DNAAF4* (NM_130810.4), c.733 C > T, p.(Arg245*), were identified through whole exome sequencing. High-speed microscopy analysis showed that most of the cilia were static in P1, with complete static of the respiratory cilia in P2. Immunofluorescence showed that the outer dynein arms (ODA) and inner dynein arms (IDA) were absent in the respiratory cilia of both probands, as well as in the sperm flagellum of P1. Transmission electron microscopy revealed the absence of ODA and IDA of respiratory cilia of P2, and HE staining showed irregular, short, absent, coiled, and bent flagella.

**Conclusion:** Our study identified a novel variant c.733C > T, which expanded the spectrum of *DNAAF4* variants. Furthermore, we linked *DNAAF4* to asthenoteratozoospermia and likely scoliosis in patients with PCD. This study will contribute to a better understanding of PCD.

## Introduction

Primary ciliary dyskinesia (PCD, MIM:244400) is a rare genetic disorder caused by motile cilia dysfunction (Mirra et al., 2017). It has diverse clinical manifestations involving multiple systems, such as recurrent respiratory infections, sinusitis, situs inversus, and infertility (Knowles et al., 2016). To date, there have been no diagnostic tests that could be completely relied upon to confirm this disease (Shapiro et al., 2016). About 50 genes have been reported as causative for PCD, of which those reported to be associated with asthenoteratozoospermia (reduced sperm motility and abnormal sperm morphology) and scoliosis are even rarer (Coutton et al., 2015; Aprea et al., 2021; Lu et al., 2021). Asthenoteratozoospermia is an important cause of male infertility, which affects approximately 7% of the male population. In addition, genetic factors account for at least 15% of the causes of male infertility (Krausz and Riera-Escamilla, 2018). Hence, finding novel genetic factors for idiopathic infertility is crucial for PCD and male infertility. In addition, scoliosis is another PCD-related phenotype reported in this article, which was defined as a spinal malformation in a patient who has skeletal maturation with a Cobb angle in the coronal plane >10° (Aebi, 2005). Scoliosis can be observed in mice with some PCD causative gene variants, but it requires more attention in patients with PCD (Baschal et al., 2015; Gao et al., 2015; Zhou et al., 2015; Lu et al., 2021).

Dynein axonemal assembly factor 4 (*DNAAF4*, OMIM: 608706), also called *DYX1C1*, encodes a 420-aa protein with a tetratricopeptide repeat domains (Taipale et al., 2003). Previous studies of *DNAAF4* focused on neuroscience and showed that it was involved in the migration of nerve cells (Taipale et al., 2003; Tapia-Páez et al., 2008). In recent years, with the in-depth research of the DNAAF family, the correlation between *DNAAF4* and motile cilia gradually emerged. DNAAF4 is localized in the cytoplasm and it was mainly involved in the preassembly of cilia multi-subunit dynein protein (Aprea et al., 2021). Variants in *DNAAF4* have been reported to be associated with bronchiectasis, situs inversus, female infertility, and abnormal ultrastructure of sperm (Guo et al., 2017; Aprea et al., 2021).

This study analyzed clinical data and whole exome sequencing data from two PCD patients, and two pathogenic variants of *DNAAF4* were identified. Thereafter, we validated the pathogenicity of the variants by high-speed microscopy, immunofluorescence, hematoxylin-eosin (HE) staining, and transmission electron microscopy (TEM). We provide the detailed report of the association of *DNAAF4* with asthenoteratozoospermia and scoliosis.

## Materials and Methods

### Patients and Clinical Materials

**FIGURE 1:**
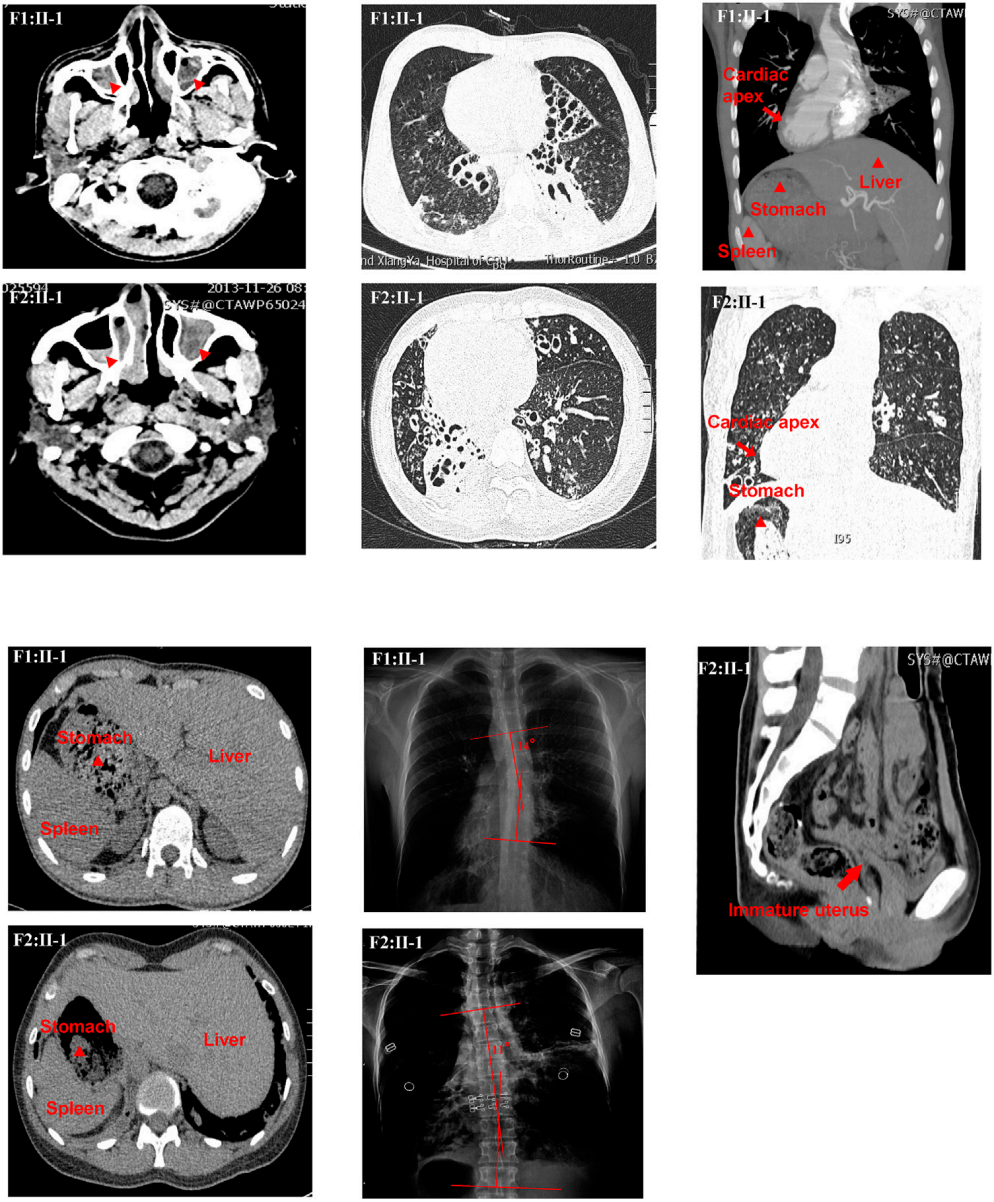
Clinical features of the two patients. The chest high-resolution computed tomography (HRCT) scan of P1 (F1:Ⅱ-1) showed bronchiectasis, rhinosinusitis, and situs inversus. Radiographic image of P1 (F1:Ⅱ-1) showed scoliosis with a Cobb’s angle of 14°. The HRCT scan of P2 (F2:Ⅱ-1) showed bronchiectasis, rhinosinusitis, situs inversus, and immature uterus. Radiographic image of P2 (F2:Ⅱ-1) showed scoliosis with a Cobb’s angle of 11°.

The study protocol was approved by the review board of the Second Xiangya Hospital of Central South University in China. Informed consent was obtained from all subjects. All experiments were performed in accordance with the relevant guidelines and regulations. Two patients diagnosed with PCD and their families were recruited, in which one patient was from a consanguineous family and the other from a non-consanguineous family. The medical records of the subjects, including pulmonary, paranasal sinuses, abdominal computed tomography (CT) (Figure 1), X-ray of the spine, and nasal nitric oxide (nNO) levels results (Supplementary Table S1) were documented and reviewed.

### Whole Exome Sequencing, Sanger Sequencing, and Bioinformatic Analysis

Blood samples from the patients and their family members were obtained with informed consent. Genomic DNA was extracted using the QIAamp DNA Blood MiniKit (250) (Qiagen, Valencia, CA) according to the manufacturer’s instructions. Whole exome capture and high-throughput sequencing were performed as previously described. In brief, genomic DNA of the patient was captured using the Agilent SureSelect Human All Exon V6 Kit (Agilent, California, United States) and sequenced on Illumina Hiseq 4000 (Illumina Inc., San Diego, United States). Following quality control, the sequencing reads were aligned to the NCBI human reference genome (GRCh37/hg19) by the Burrows Wheeler Aligner. ANNOVAR was employed to annotate the variant call format file.

Single-nucleotide variants (SNVs) and short insertions and deletions (indels) were filtered as follows: 1) Retain rare variants (minor allele frequency <0.01) in 1,000 Genomes Project (1,000G), NHLBI-ESP project, Exome Aggregation Consortium (ExAC4), and in-house database of Novogene. 2) Noncoding and intronic variants were filtered. 3) Synonymous missense variants were excluded. 4) Bioinformatics analyses (SIFT, Polyphen-2, MutationTaster, MutationAssessor, and CADD) were used for the remaining variants. 5) A PCD or PCD-candidate gene list derived from literatures was used to identify the disease-associated variant, as described in our previous study (Lei et al., 2022).

Sanger sequencing was used to validate the variant in the individuals. Primers were designed using an online tool (PrimerQuest, IDT, https://sg.idtdna.com/PrimerQuest). The primer sequences were as follows: proband 1, forward 5′- GCC​CAT​CCC​TGA​GTC​AAT​TA -3′, reverse 5′-GAG​ACC​TGC​CTG​TGC​AAT​A-3’; proband 2, forward 5′-GTG​ACT​TGT​TTG​CTA​CCA​TTG​TT-3′, reverse 5′-AGT​CGG​TAT​TCT​CTT​ATC​ACT​ATT​CTG-3′. PCR products were sequenced by the ABI PRISM 3730 DNA Analyzer using the BigDye Terminator v3.1 Cycle Sequencing Kit.

Evolutionary conservation analysis was performed by aligning the amino acid sequences of DNAAF4 proteins from different vertebrate species obtained from the GenBank database (https://www.ncbi.nlm.nih.gov/homologene/).

### Transmission Electron Microscopy

To understand the abnormalities in the ultrastructure of the respiratory cilia, cilia were examined by means of electron microscopy. Samples of nasal mucosa (F2: II-1 and control) were fixed in 2.5% glutaraldehyde in 0.1 M sodium cacodylate buffer at 4°C, washed overnight, and postfixed in 1% osmium tetroxide. Following dehydration, the samples were embedded in epoxy resin. Following polymerization, several sections were picked out onto copper grids. The sections were stained with aqueous 1% uranyl acetate and Reynold’s lead citrate. An AHT7700 Hitachi electron microscope (Hitachi, Tokyo, Japan) and a MegaView Iii digital camera (Olympus Soft Imaging Solutions GmbH, Münster, Germany) were used to capture images.

### Immunofluorescence Analysis

Nasal brushing biopsy samples (F1: II-1, F2: II-1, and control) and sperm specimens (F1: II-1 and control) were collected. For immunofluorescence analysis, the slides were incubated with primary antibodies (DNAH5, DNALI1, DNAI1, and anti-acetylated tubulin monoclonal antibody, respectively) for 2.5 h at 37°C. The slides were then incubated with secondary antibodies [Alexa Fluor^®^ 488 antimouse IgG (A-21121) and Alexa Fluor^®^ 555 anti-rabbit IgG (A31572)] for 1.5 h at 37°C. We used anti-acetylated tubulin monoclonal antibody (T7451, 1:500, Sigma-Aldrich, Missouri, United States) to mark the ciliary axonemal, DNAH5 (HPA037470, 1:25, Sigma-Aldrich, Missouri, United States) to label the outer dynein arm (ODA), DNALI1 (HPA028305, 1:25, Sigma-Aldrich, Missouri, United States) to label the inner dynein arm (IDA) of respiratory mucosa, DNAI1 (ab171964,1:25, Abcam, Cambridgeshire, United Kingdom) to outer dynein arm (ODA) of sperm, and DAPI to label the nuclei. An upright Olympus BX53 microscope (Olympus, Tokyo, Japan) and cellSens Dimension software (Olympus, Tokyo, Japan) were utilized to photograph fluorescence signals.

### High-Speed Video Microscopy Analysis

Nasal brush biopsy samples (F1: II-1, F2: II-1, and control) were suspended in Gibco Medium 199 (12350039 Gibco). Strips of ciliated epithelium were imaged using an upright Olympus BX53 microscope (Olympus, Tokyo, Japan) with a 40x objective lens. Videos were recorded using a scientific complementary metal oxide semiconductor camera (Prime BSI, Teledyne Photometrics Inc., United States) at a rate of 500 frames per second (fps) at room temperature. Intact ciliated edge >50 μm was used for functional analysis only.

## Results

### Clinical Summary

Proband 1 (P1, F1: II-1), a 19-year-old man, comes from a nonconsanguineous family-1. He had typical PCD symptoms, such as chronic cough and sputum production, dyspnea, hemoptysis, sinusitis, etc. In addition, he complained of abnormalities in his sense of smell and hearing. However, his fertility was unknown because he was not married. He denied any history of asthma, tuberculosis, measles pneumonia, or drowning. No relevant family history was reported. High-resolution CT (HRCT) showed sinusitis, bronchiectasis, and situs inversus. Upright radiograph revealed scoliosis with a Cobb’s angle of 14° (Figure 1). Furthermore, the semen routine test showed that the percentage of motile sperm was 0. Proband 2 (P2, F2: II-1), a 37-year-old woman, comes from a consanguineous family-2. She had chronic cough and sputum, dyspnea, and sinusitis. She also had abnormalities in the sense of smell and hearing. In addition, she suffered from infertility. HRCT findings were similar to P1, and upright radiograph revealed scoliosis with a Cobb’s angle of 11° (Figure1). The HRCT also indicated an immature uterus. Her forced expiratory volume in 1 second (FEV1)%prediction, forced vital capacity (FVC)%prediction, FEV1/FVC, and nasal NO were 36%, 50%, 60%, and 8.4 nL/min, respectively (Supplementary Tables S1–S3; Figure1).

### Whole Exome Sequencing Identified the *DNAAF4* Variants

**FIGURE 2:**
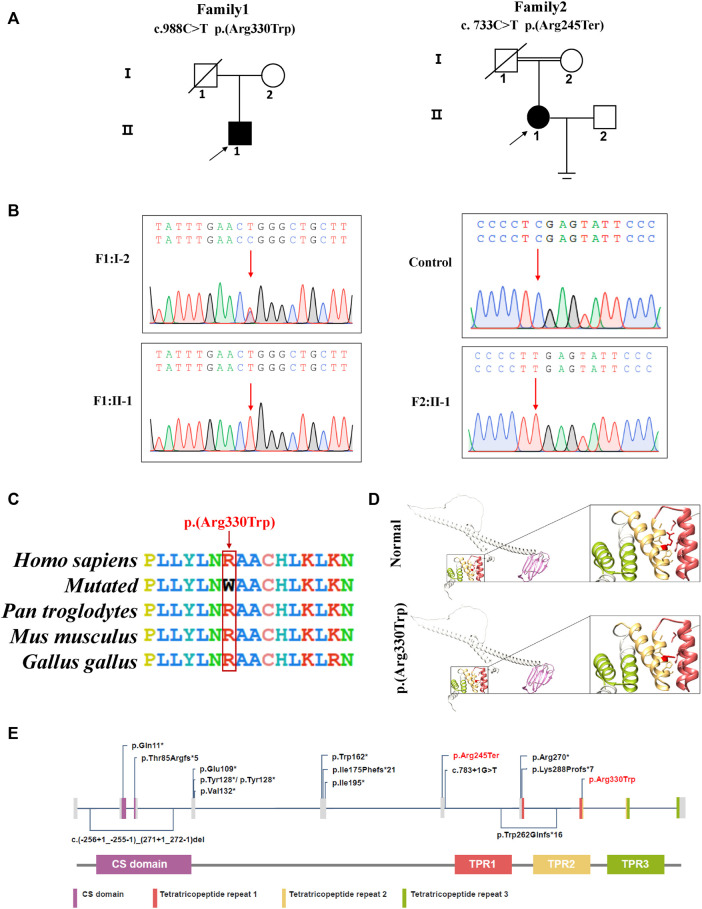
*DNAAF4* variants were identified in two patients with PCD. **(A)** pedigree analysis of the two patients from two Han Chinese families, where family 2 is a consanguineous family. Circles indicate women. Squares indicate men. Solid symbols indicate patients. Crossed-out symbols mean that subjects had passed away. The arrows indicate the probands. **(B)** the two variants were validated by Sanger sequencing. Red arrows indicate the variant sites; **(C)** conservative analysis of the missense variant **(C)** 988C > T (p.Arg330Trp). Red box indicates mutant amino acid sites. **(D)** 3D mock structure of DNAAF4. The purple region is the CS domain, while green, yellow, and brick red indicate the three tetratricopeptide repeats, respectively. The variant p.(Arg330Trp) is indicated by red color. **(E)** DNAAF4 protein structure and the reported disease-causing variants of DNAAF4. Variants reported in this study are highlighted in red.

We used the patient’s peripheral blood to extract DNA for Whole exome sequencing (WES). After WES, 12.02 and 6.8 GB data were used for subsequent analysis. The percentages of bases with a coverage depth of more than 10× were 99.6% and 98.1%, respectively. Two variants, *DNAAF4* (NM_130810.4): c.988C > T (p.Arg330Trp) in P1 and a novel variant *DNAAF4* (NM_130810.4): c.733C > T (p.Arg245Ter) in P2, were identified. We classified the pathogenicity of the two variants according to ACMG (Richards et al., 2015). The results showed the variant in P1 was consistent with PM2+PM3+PP3+PP5 (likely pathogenic), and the variant in P2 met the criteria PVS1+PM2+PP5 (pathogenic). These two disease-causing variants were validated by Sanger sequencing (Supplementary Table S2; Figure2).

### Experimental Validation of the Pathogenicity of Variants

**FIGURE 3:**
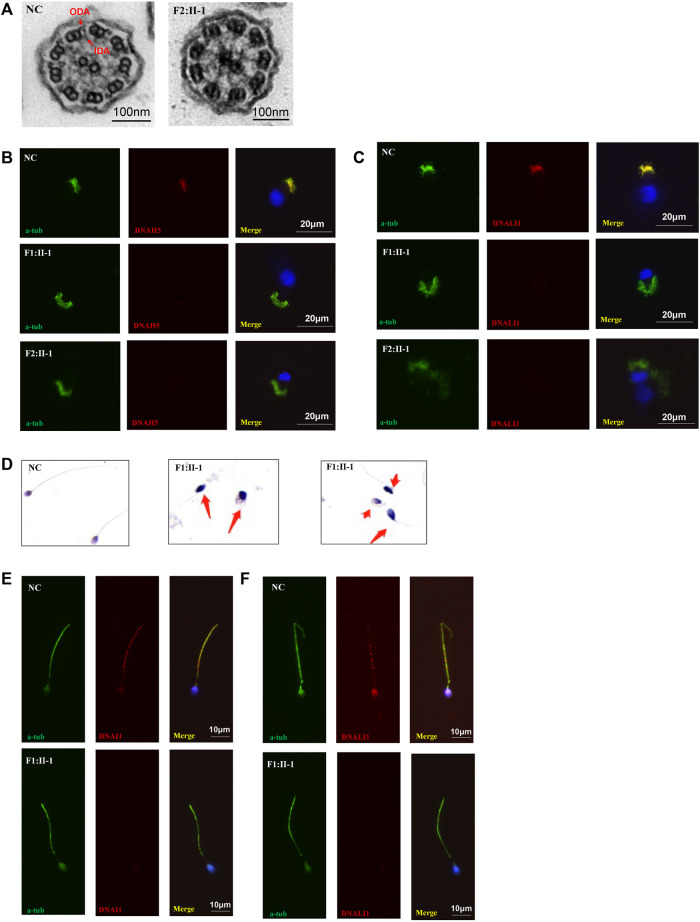
TEM, HE staining, and immunofluorescence analysis of the nasal ciliated cells and sperm. **(A)** TEM analysis indicated loss of IDAs and ODAs of P2 (F1:Ⅱ-1) compared with the normal control. Scale bars, 100 nm; **(B,C)** immunofluorescence of nasal ciliated cells revealed the absence of DNAH5 and DNALI1 (red) of the two patients compared with normal control. Anti-acetylated tubulin monoclonal antibody was used to mark the ciliary axoneme. DNAH5 was used to label the outer dynein arm (ODA), DNALI1 was used to label the inner dynein arm (IDA), and DAPI was used to label the nuclei. Scale bars, 20 μm; **(D)** HE staining of sperm. Compared to normal controls, the patient’s sperm had significant short, coiled, and irregular tails. **(E)** immunofluorescence analysis showed the absence of DNALI1 and DNAI1 (red) in the mutant sperm from P1 (F1: II-1) compared with the normal control. Scale bars, 10 μm.

High-speed microscopy showed complete static of sperm flagella and a few residual beating of respiratory cilia in P1 and complete static of the respiratory cilia in P2 compared with normal control (Supplementary Videos S1–S3). Immunofluorescence showed the deficiency of DNAH5 and DNALI1, which labelled ODA and IDA, respectively, in the respiratory cilia of both probands. In addition, the DNALI1 and DNAI1 which respectively labelled the ODA and IDA in the sperm flagellum of P1 were also absent. TEM revealed the absence of ODA and IDA of P2 respiratory cilia. HE staining showed irregular flagella, short, absent, coiled, and bent, indicating asthenoteratozoospermia (Figure3).

## Discussion

In this study, whole exome sequencing and Sanger sequencing were used to identify and confirm homozygous variants in *DNAAF4*. The pathogenicity of the variants was validated by high-speed microscopy, immunofluorescence, HE staining, and TEM. Both patients were infertile, one of whom was diagnosed with asthenoteratozoospermia. In addition, the upright radiograph suggested scoliosis in both cases.

PCD is a genetic disorder caused by motile cilia dysfunction (Lucas et al., 2020). Motile cilia are hair-like structures. The ultrastructure of most motile cilia shows that they are composed of nine peripheral duplex microtubules, two central single microtubules and corresponding spokes (9 + 2 structure) (Fliegauf et al., 2007). Flagella have a similar structure. In addition, there are many multisubunit motor protein complexes within the cilia and flagella, such as outer dynein arms (ODA) and inner dynein arms (IDA) that attach to the peripheral microtubules. They are essential for the generation, and the regulation of cilia and flagella beating (Fliegauf et al., 2007; Aprea et al., 2021).

*DNAAF4* is located on Chromosome 15 with 10 exons encoding a 420 aa protein. It is associated with the preassembly of axonemal arms and is involved in the integration and stabilization of the intermediate chain, which is an integral component of both the ODA and IDA (Tarkar et al., 2013; Aprea et al., 2021). Here, we have reviewed the literature and summarized the 15 PCD-associated variants in *DNAAF4* (Figure 2E; Supplementary Table S4) (Tarkar et al., 2013; Marshall et al., 2015; Guo et al., 2017; Ceyhan-Birsoy et al., 2019; Olm et al., 2019; Blanchon et al., 2020; Aprea et al., 2021). Most studies focused on the respiratory phenotype of patients, and a small number of studies reported situs inversus. Moreover, few DNAAF4-related studies reported the details of the sperm phenotype (Tarkar et al., 2013; Guo et al., 2017; Aprea et al., 2021). Both our patients with *DNAAF4* variants had situs inversus, and the male patient had asthenoteratozoospermia. In these two cases, two homozygous *DNAAF4* variants were reported in this study, and the pathogenicity of the variants was validated by high-speed microscopy, immunofluorescence, HE staining, and TEM. *DNAAF4* c.988C > T leads to immotility of most respiratory cilia and complete static of sperm flagella. HE staining indicated typical teratozoospermic. Although this variant has been reported before, only electron microscopic findings of respiratory cilia have been described. No observations were made on sperm phenotype. Furthermore, previously reported patient did not have situs inversus (Marshall et al., 2015). *DNAAF4* c.733 C > T results in completely respiratory cilia static. In both cases, the respiratory cilia showed the absence of ODA and IDA. Furthermore, in the male patient, both ODA and IDA of the sperm flagellum were absent.

Although asthenoteratozoospermia has been proposed for a long time, it is only recently that the research has focused on the contribution of abnormal sperm ultrastructure to pathogenesis (Sandler, 1952). Abnormalities in the development of the axoneme and its accessory structures are thought to be the main cause of asthenoteratozoospermia (Jiao et al., 2021). Asthenoteratozoospermia was previously described separately as asthenozoospermia and teratozoospermia. When the percentage of motile sperm in the semen is <40%, it is called asthenozoospermia. Teratozoospermia means that the spermatozoa of patients with deformed spermatozoa including defects in the head, midpiece, and/or tail (tend to be short, coiled, absent, or irregular flagella) (Ben Khelifa et al., 2014). Teratozoospermia is currently diagnosed by light microscopic staining or scanning electron microscopy of sperm morphology and asthenozoospermia by high-speed microscope (WHO, 2021). They can severely reduce male fertility, but have received less attention in previous PCD studies (Dávila Garza and Patrizio, 2013; Sironen et al., 2020). Although >50 PCD-associated pathogenic genes have been reported, a few have been reported to be associated with asthenoteratozoospermia, such as *DNAH9*, *DNAH1*, *ARMC4*, *BRWD1*, *CCDC39*, *CCDC40*, *DRC1*, *CFAP74*, and *SPEF2* (Neesen et al., 2001; Sha et al., 2020; Tu et al., 2020; Chen et al., 2021; Gao et al., 2021; Guo et al., 2021; Lei et al., 2022; Xu et al., 2022). In most other cases, researchers have not analyzed the semen of male patients. Despite advances in male infertility testing, in 40% of patients, it was not possible to identify the cause. Given that the sperm flagellum has a similar structure to the respiratory cilia, semen analysis in men with PCD can help identify the cause of male infertility and thus provide a reference for assisted reproduction (Krausz and Riera-Escamilla, 2018).

In addition, both patients had the classic PCD phenotype as well as the more commonly overlooked scoliosis. Although scoliosis is currently reported less commonly in patients with PCD, this phenotype is more frequently observed in model organisms carrying the causative gene for PCD, including *CCDC40*, *DNAAF4*, *ODAD3*, and *ZMYND10* (Grimes et al., 2016; Kobayashi et al., 2017; Lu et al., 2021). A previous study evaluated chest radiographs in 198 patients with PCD and found a Cobb angle >10° in 8% of patients, but that study did not perform genetic analysis (Schlösser et al., 2017). The etiology of idiopathic scoliosis is not well understood, and different hypotheses have been proposed (Aebi, 2005; Cheng et al., 2015; Oliazadeh et al., 2017). Of these, abnormal cilia function has been suggested as a possible cause. Researchers believed that “sensory organs,” including motor cilia, neurons in contact with the cerebrospinal fluid, Reissner fibers, and Urotensin neuropeptide signals, were essential for the proper development of the perceptual axis (Bearce and Grimes, 2021). An alternative hypothesis is that scoliosis in patients with PCD is because of situs inversus (Schlösser et al., 2017). However, scoliosis is often overlooked in patients with PCD as anteroposterior and lateral radiographs of the spine are not a routine examination. Although there are few reports linking scoliosis to PCD, some genes have been reported to be associated with abnormal cilia beating and scoliosis in previous research (Fei et al., 2010; Makrythanasis et al., 2014; Baschal et al., 2015; Zhou et al., 2015; Lu et al., 2021; Nita et al., 2021). As an increasing number of PCD model organisms are observed with scoliosis, the spinal examination of the patient needs to be taken seriously.

It should be noted that the female patient reported here also had an infantile uterus. Therefore, the effect of the variant on female fertility caused by dysfunctional cilia in fallopian tubes cannot be determined. Furthermore, as scoliosis is currently less commonly reported in PCD-related human genetic studies, the effect of *DNAAF4* variants on the human spine needs further validation (Muñoz-Montecinos et al., 2021).

Overall, we identified two homozygous variants in the PCD-associated gene *DNAAF4*, c.988C > T and c.733 C > T, which expand the genetic spectrum of PCD. In particular, we provide additional references for asthenoteratozoospermia and scoliosis in patients with PCD, which has previously received less attention.

## Data Availability

The datasets for this article are not publicly available due to concerns regarding participant/patient anonymity. Requests to access the datasets should be directed to the corresponding author.

## References

[B1] Aebi M. (2005). The Adult Scoliosis. Eur. Spine J. 14, 925–948. 10.1007/s00586-005-1053-9 16328223

[B2] Aprea I. Raidt J. Höben I. M. Loges N. T. Nöthe-Menchen T. Pennekamp P. (2021). Defects in the Cytoplasmic Assembly of Axonemal Dynein Arms Cause Morphological Abnormalities and Dysmotility in Sperm Cells Leading to Male Infertility. PLoS Genet. 17, e1009306. 10.1371/journal.pgen.1009306 33635866 PMC7909641

[B3] Baschal E. E. Swindle K. Justice C. M. Baschal R. M. Perera A. Wethey C. I. (2015). Sequencing of the TBX6 Gene in Families with Familial Idiopathic Scoliosis. Spine Deform. 3, 288–296. 10.1016/j.jspd.2015.01.005 26120555 PMC4480874

[B4] Bearce E. A. Grimes D. T. (2021). On Being the Right Shape: Roles for Motile Cilia and Cerebrospinal Fluid Flow in Body and Spine Morphology. Seminars Cell Dev. Biol. 110, 104–112. 10.1016/j.semcdb.2020.07.005 32693941

[B5] Ben Khelifa M. Coutton C. Zouari R. Karaouzène T. Rendu J. Bidart M. (2014). Mutations in DNAH1, Which Encodes an Inner Arm Heavy Chain Dynein, Lead to Male Infertility from Multiple Morphological Abnormalities of the Sperm Flagella. Am. J. Hum. Genet. 94, 95–104. 10.1016/j.ajhg.2013.11.017 24360805 PMC3882734

[B6] Blanchon S. Legendre M. Bottier M. Tamalet A. Montantin G. Collot N. (2020). Deep Phenotyping, Including Quantitative Ciliary Beating Parameters, and Extensive Genotyping in Primary Ciliary Dyskinesia. J. Med. Genet. 57, 237–244. 10.1136/jmedgenet-2019-106424 31772028

[B7] Ceyhan-Birsoy O. Murry J. B. Machini K. Lebo M. S. Yu T. W. Fayer S. (2019). Interpretation of Genomic Sequencing Results in Healthy and Ill Newborns: Results from the BabySeq Project. Am. J. Hum. Genet. 104, 76–93. 10.1016/j.ajhg.2018.11.016 30609409 PMC6323417

[B8] Chen D. Liang Y. Li J. Zhang X. Zheng R. Wang X. (2021). A Novel CCDC39 Mutation Causes Multiple Morphological Abnormalities of the Flagella in a Primary Ciliary Dyskinesia Patient. Reprod. Biomed. Online 43, 920–930. 10.1016/j.rbmo.2021.07.005 34674941

[B9] Cheng J. C. Castelein R. M. Chu W. C. Danielsson A. J. Dobbs M. B. Grivas T. B. (2015). Adolescent Idiopathic Scoliosis. Nat. Rev. Dis. Prim. 1, 15030. 10.1038/nrdp.2015.30 27188385

[B10] Coutton C. Escoffier J. Martinez G. Arnoult C. Ray P. F. (2015). Teratozoospermia: Spotlight on the Main Genetic Actors in the Human. Hum. Reprod. Update 21, 455–485. 10.1093/humupd/dmv020 25888788

[B11] Dávila Garza S. A. Patrizio P. (2013). Reproductive Outcomes in Patients with Male Infertility Because of Klinefelter's Syndrome, Kartagener's Syndrome, Round-Head Sperm, Dysplasia Fibrous Sheath, and 'stump' Tail Sperm. Curr. Opin. Obstet. Gynecol. 25, 229–246. 10.1097/gco.0b013e32835faae5 23587797

[B12] Fei Q. Wu Z. Wang H. Zhou X. Wang N. Ding Y. (2010). The Association Analysis of TBX6 Polymorphism with Susceptibility to Congenital Scoliosis in a Chinese Han Population. Spine 35, 983–988. 10.1097/brs.0b013e3181bc963c 20228709

[B13] Fliegauf M. Benzing T. Omran H. (2007). When Cilia Go Bad: Cilia Defects and Ciliopathies. Nat. Rev. Mol. Cell Biol. 8, 880–893. 10.1038/nrm2278 17955020

[B14] Gao C. Chen B. P. Sullivan M. B. Hui J. Ouellet J. A. Henderson J. E. (2015). Micro CT Analysis of Spine Architecture in a Mouse Model of Scoliosis. Front. Endocrinol. 6, 38. 10.3389/fendo.2015.00038 PMC4365746 25852647

[B15] Gao Y. Xu C. Tan Q. Shen Q. Wu H. Lv M. (2021). Case Report: Novel Biallelic Mutations in ARMC4 Cause Primary Ciliary Dyskinesia and Male Infertility in a Chinese Family. Front. Genet. 12, 715339. 10.3389/fgene.2021.715339 34394199 PMC8362595

[B16] Grimes D. T. Boswell C. W. Morante N. F. C. Henkelman R. M. Burdine R. D. Ciruna B. (2016). Zebrafish Models of Idiopathic Scoliosis Link Cerebrospinal Fluid Flow Defects to Spine Curvature. Science 352, 1341–1344. 10.1126/science.aaf6419 27284198 PMC5574193

[B17] Guo T. Tan Z.-P. Chen H.-M. Zheng D.-y. Liu L. Huang X.-G. (2017). An Effective Combination of Whole-Exome Sequencing and Runs of Homozygosity for the Diagnosis of Primary Ciliary Dyskinesia in Consanguineous Families. Sci. Rep. 7, 7905. 10.1038/s41598-017-08510-z 28801648 PMC5554225

[B18] Guo T. Tu C.-F. Yang D.-H. Ding S.-Z. Lei C. Wang R.-C. (2021). Bi-allelic BRWD1 Variants Cause Male Infertility with Asthenoteratozoospermia and Likely Primary Ciliary Dyskinesia. Hum. Genet. 140, 761–773. 10.1007/s00439-020-02241-4 33389130

[B19] Jiao S.-Y. Yang Y.-H. Chen S.-R. (2021). Molecular Genetics of Infertility: Loss-Of-Function Mutations in Humans and Corresponding Knockout/mutated Mice. Hum. Reprod. Update 27, 154–189. 10.1093/humupd/dmaa034 33118031

[B20] Knowles M. R. Zariwala M. Leigh M. (2016). Primary Ciliary Dyskinesia. Clin. Chest Med. 37, 449–461. 10.1016/j.ccm.2016.04.008 27514592 PMC4988337

[B21] Kobayashi D. Asano-Hoshino A. Nakakura T. Nishimaki T. Ansai S. Kinoshita M. (2017). Loss of Zinc Finger MYND-type Containing 10 (Zmynd10) Affects Cilia Integrity and Axonemal Localization of Dynein Arms, Resulting in Ciliary Dysmotility, Polycystic Kidney and Scoliosis in Medaka (*Oryzias latipes*). Dev. Biol. 430, 69–79. 10.1016/j.ydbio.2017.08.016 28823919

[B22] Krausz C. Riera-Escamilla A. (2018). Genetics of Male Infertility. Nat. Rev. Urol. 15, 369–384. 10.1038/s41585-018-0003-3 29622783

[B23] Lei C. Yang D. Wang R. Ding S. Wang L. Guo T. (2022). DRC1 Deficiency Caused Primary Ciliary Dyskinesia and MMAF in a Chinese Patient. J. Hum. Genet. 67, 197–201. 10.1038/s10038-021-00985-z 34815526

[B24] Lu C. Yang D. Lei C. Wang R. Guo T. Luo H. (2021). Identification of Two Novel DNAAF2 Variants in Two Consanguineous Families with Primary Ciliary Dyskinesia. Pharmgenomics Pers. Med. 14, 1415–1423. 10.2147/pgpm.s338981 34785929 PMC8591118

[B25] Lucas J. S. Davis S. D. Omran H. Shoemark A. (2020). Primary Ciliary Dyskinesia in the Genomics Age. Lancet Respir. Med. 8, 202–216. 10.1016/s2213-2600(19)30374-1 31624012

[B26] Makrythanasis P. Temtamy S. Aglan M. S. Otaify G. A. Hamamy H. Antonarakis S. E. (2014). A Novel Homozygous Mutation in FGFR3 Causes Tall Stature, Severe Lateral Tibial Deviation, Scoliosis, Hearing Impairment, Camptodactyly, and Arachnodactyly. Hum. Mutat. 35, 959–963. 10.1002/humu.22597 24864036

[B27] Marshall C. R. Scherer S. W. Zariwala M. A. Lau L. Paton T. A. Stockley T. (2015). Whole-Exome Sequencing and Targeted Copy Number Analysis in Primary Ciliary Dyskinesia. G3 (Bethesda) 5, 1775–1781. 10.1534/g3.115.019851 26139845 PMC4528333

[B28] Mirra V. Werner C. Santamaria F. (2017). Primary Ciliary Dyskinesia: An Update on Clinical Aspects, Genetics, Diagnosis, and Future Treatment Strategies. Front. Pediatr. 5, 135. 10.3389/fped.2017.00135 28649564 PMC5465251

[B29] Muñoz-Montecinos C. Romero A. Sepúlveda V. Vira M. Fehrmann-Cartes K. Marcellini S. (2021). Turning the Curve into Straight: Phenogenetics of the Spine Morphology and Coordinate Maintenance in the Zebrafish. Front. Cell Dev. Biol. 9, 801652. 10.3389/fcell.2021.801652 35155449 PMC8826430

[B30] Neesen J. Kirschner R. Ochs M. Schmiedl A. Habermann B. Mueller C. (2001). Disruption of an Inner Arm Dynein Heavy Chain Gene Results in Asthenozoospermia and Reduced Ciliary Beat Frequency. Hum. Mol. Genet. 10, 1117–1128. 10.1093/hmg/10.11.1117 11371505

[B31] Nita A. Abraham S. P. Krejci P. Bosakova M. (2021). Oncogenic FGFR Fusions Produce Centrosome and Cilia Defects by Ectopic Signaling. Cells 10, 1445. 10.3390/cells10061445 34207779 PMC8227969

[B32] Oliazadeh N. Gorman K. F. Eveleigh R. Bourque G. Moreau A. (2017). Identification of Elongated Primary Cilia with Impaired Mechanotransduction in Idiopathic Scoliosis Patients. Sci. Rep. 7, 44260. 10.1038/srep44260 28290481 PMC5349607

[B33] Olm M. A. K. Marson F. A. L. Athanazio R. A. Nakagawa N. K. Macchione M. Loges N. T. (2019). Severe Pulmonary Disease in an Adult Primary Ciliary Dyskinesia Population in Brazil. Sci. Rep. 9, 8693. 10.1038/s41598-019-45017-1 31213628 PMC6582273

[B34] Richards S. Aziz N. Bale S. Bick D. Das S. Gastier-Foster J. (2015). Standards and Guidelines for the Interpretation of Sequence Variants: A Joint Consensus Recommendation of the American College of Medical Genetics and Genomics and the Association for Molecular Pathology. Genet. Med. 17, 405–424. 10.1038/gim.2015.30 25741868 PMC4544753

[B35] Sandler B. (1952). The Relation of Cervical Mucus and Asthenospermia in Sterility. J. Obstet. Gynaecol. Br. Emp. 59, 202–207. 10.1111/j.1471-0528.1952.tb04115.x 14928088

[B36] Schlösser T. P. C. Semple T. Carr S. B. Padley S. Loebinger M. R. Hogg C. (2017). Scoliosis Convexity and Organ Anatomy Are Related. Eur. Spine J. 26, 1595–1599. 10.1007/s00586-017-4970-5 28180983

[B37] Sha Y. Wei X. Ding L. Ji Z. Mei L. Huang X. (2020). Biallelic Mutations of CFAP74 May Cause Human Primary Ciliary Dyskinesia and MMAF Phenotype. J. Hum. Genet. 65, 961–969. 10.1038/s10038-020-0790-2 32555313

[B38] Shapiro A. J. Zariwala M. A. Ferkol T. Davis S. D. Sagel S. D. Dell S. D. (2016). Diagnosis, Monitoring, and Treatment of Primary Ciliary Dyskinesia: PCD Foundation Consensus Recommendations Based on State of the Art Review. Pediatr. Pulmonol. 51, 115–132. 10.1002/ppul.23304 26418604 PMC4912005

[B39] Sironen A. Shoemark A. Patel M. Loebinger M. R. Mitchison H. M. (2020). Sperm Defects in Primary Ciliary Dyskinesia and Related Causes of Male Infertility. Cell. Mol. Life Sci. 77, 2029–2048. 10.1007/s00018-019-03389-7 31781811 PMC7256033

[B40] Taipale M. Kaminen N. Nopola-Hemmi J. Haltia T. Myllyluoma B. Lyytinen H. (2003). A Candidate Gene for Developmental Dyslexia Encodes a Nuclear Tetratricopeptide Repeat Domain Protein Dynamically Regulated in Brain. Proc. Natl. Acad. Sci. U.S.A. 100, 11553–11558. 10.1073/pnas.1833911100 12954984 PMC208796

[B41] Tapia-Páez I. Tammimies K. Massinen S. Roy A. L. Kere J. (2008). The Complex of TFII-I, PARP1, and SFPQ Proteins Regulates the DYX1C1 Gene Implicated in Neuronal Migration and Dyslexia. FASEB J. 22, 3001–3009. 10.1096/fj.07-104455 18445785 PMC2493457

[B42] Tarkar A. Loges N. T. Loges N. T. Slagle C. E. Francis R. Dougherty G. W. (2013). DYX1C1 is Required for Axonemal Dynein Assembly and Ciliary Motility. Nat. Genet. 45, 995–1003. 10.1038/ng.2707 23872636 PMC4000444

[B43] Tu C. Nie H. Meng L. Wang W. Li H. Yuan S. (2020). Novel Mutations in SPEF2 Causing Different Defects between Flagella and Cilia Bridge: The Phenotypic Link between MMAF and PCD. Hum. Genet. 139, 257–271. 10.1007/s00439-020-02110-0 31942643

[B44] WHO (2021). WHO Laboratory Manual for the Examination and Processing of Human Semen. sixth edition. Geneva: World Health Organization. 10.5534/wjmh.210074 PMC8443989 34169684

[B45] Xu Y. Yang B. Lei C. Yang D.-h. Ding S. Lu C. (2022). Novel Compound Heterozygous Variants in CCDC40 Associated with Primary Ciliary Dyskinesia and Multiple Morphological Abnormalities of the Sperm Flagella. Pharmgenomics Pers. Med. 15, 341–350. 10.2147/pgpm.s359821 35449766 PMC9017783

[B46] Zhou S. Xie Y. Tang J. Huang J. Huang Q. Xu W. (2015). FGFR3 Deficiency Causes Multiple Chondroma-like Lesions by Upregulating Hedgehog Signaling. PLoS Genet. 11, e1005214. 10.1371/journal.pgen.1005214 26091072 PMC4474636

