# Supplementary material for: Case Report: DNAAF4 Variants Cause Primary Ciliary Dyskinesia and Infertility in Two Han Chinese Families
Source: Front Genet. 2022 Jul 12;13:934920. doi: 10.3389/fgene.2022.934920 (PMC9315306; doi:10.3389/fgene.2022.934920)
Supplement: Supplementary file 3 [file DataSheet1.doc]

Table S1 Summary of the Clinical Features of Two Patients with PCD

| **Characteristics** | **F1:Ⅱ-1** | **F2:Ⅱ-1** |
| --- | --- | --- |
| Sex | Male | Female |
| Age (years) | 19 | 37 |
| Onset | Childhood | Childhood |
| Consanguinity | No | Yes |
| Inbreeding coefficient | NA | 1/16 |
| CT | Bronchiectasis  Rhinosinusitis  Visceral inversion | Bronchiectasis  Rhinosinusitis  Visceral inversion  Immature uterus |
| Fertility problems | Unknown | Yes |
| Smell problem | Yes | Yes |
| Hearing problems | Yes | Yes |
| Comorbidities | Scoliosis; Bronchial arteriovenous fistula | Scoliosis; Congenital uterine malformation，Immature uterus |
| FEV1%prediction | NA | 36% |
| FVC%prediction | NA | 50% |
| FEV1/FVC | NA | 60% |
| Cobb’s angle | 14° | 11° |
| Nasal NO (nL/min) | NA | 8.4 |

Abbreviation: NA, not available.

Table S2 Variants identified by whole-exome sequencing in combination with PCD-related gene-filtering for the patients

| **Patient** | **Gene** | **Position** | **RefSeq ID** | **Amino Acid Alteration** | **Function** | **1000 G** | **GO-ESP** | **ExAC** | **MutationTaster** | **SIFT** | **Poly-Phen2** | **CADD** | **ACMG** |
| --- | --- | --- | --- | --- | --- | --- | --- | --- | --- | --- | --- | --- | --- |
| F1:Ⅱ-1 | *DNAAF4* | Chr15： 55727162 | NM_130810.4 | c.988C>T, p.(Arg330Trp) | Missense | NA | NA | 0.00002473 | D | D | D | 34 | PM2+PM3+PP3+PP5 |
| F2:Ⅱ-1 | *DNAAF4* | Chr15: 55742470 | NM_130810.4 | c.733C>T, p.(Arg245Ter) | stopgain | NA | NA | 0.00000824 | A | NA | NA | 42 | PVS1+PM2+PP5 |

Abbreviation: NA, not available. D, Disease causing. A, Disease causing automatic.

Table S3 Semen routine examination of the PCD patient（F1: II-1）

| **Semen parameters** | **P1（F1: II-1）** | **Normal control** | **Normal spermic parameters** |
| --- | --- | --- | --- |
| Sperm volume (mL) | 2.6 | 2.9 | ≥1.5ml |
| Sperm concentration (million/mL) | 20.6 | 56 | ≥ 15 |
| Motile sperm (%) | 0 | 52 | ≥ 40 |
| Vitality (%) | 70 | 72 | ≥ 58 |

Table S4 Clinical phenotypes in patients with reported *DNAAF4* variants.

|  | Variants | Age  (years) | Gender | HSVA (resp.cells) | nNO  (nL/min) | Sistus Inversus | Sinusitis | Infertility | IF defect  (resp. cells) | IF defect  (sperm) | TEM defect (resp.cells) | Reference |
| --- | --- | --- | --- | --- | --- | --- | --- | --- | --- | --- | --- | --- |
| Case 1 | c.31 C>T,  p.Gln11* | NA | male | immotile | 12.7 | yes | NA | yes | DNAI1,  DNAI2,  DNALI1  absent | DNAI1, DNAI2, DNALI1, DNAH8, DNAH17 abnormal | ODA | Aprea (2021) PLoS Genet 17, |
| Case 2 | c.325G>T,  p.Glu109* | 31 | male | NA | NA | no | yes | yes | NA | NA | ODA+IDA | Tarkar (2013) Nat Genet 45, 995 |
| Case 3 | c.384C>A,  p.Try128*;  c.485G>A,  p.Trp162* | 18 | male | NA | NA | yes | no | NA | NA | NA | ODA+IDA | Tarkar (2013) Nat Genet 45, 995 |
| Case 4 | c.808C>T,  p.270Arg* | 8 | female | immotile | NA | situs ambiguus | yes | NA | NA | NA | ODA+IDA | Tarkar (2013) Nat Genet 45, 995 |
| Case 5 | c.988C>T,  p.Arg330Trp | 4 | male | NA | 13.4 | no | NA | NA | NA | NA | ODA+IDA | Marshall (2015) G3 (Bethesda) 5, 1775 |
| Case 6 | c.783+1G>T | 73 | female | NA | NA | no | yes | yes | NA | NA | ODA+IDA | Tarkar (2013) Nat Genet 45, 995 |
| Case 7 | c.384delC,  p.Tyr128* | 41 | male | NA | 5.0 | yes | yes | yes | NA | NA | NA | Guo (2017) Sci Rep 7, 7905 |
| Case 8 | c.390_393delAAGT,  p.Val132* | 43 | male | immotile | NA | no | yes | yes | NA | NA | ODA+IDA | Tarkar (2013) Nat Genet 45, 995 |
| Case 9 | c.523delA,  p.Ile175Phefs*21 | NA | NA | NA | NA | NA | NA | NA | NA | NA | NA | Ceyhan-Birsoy (2019) Am J Hum Genet 104, 76 |
| Case 10 | c.583delA,  p.Ile195* | 15 | male | partially immotile | NA | yes | NA | NA | NA | NA | NA | Tarkar (2013) Nat Genet 45, 995 |
| Case 11 | c.862_866delAAGAA,  p.Lys288Profs*7 | 25 | female | NA | NA | yes | NA | NA | NA | NA | ODA+IDA | Olm (2019) Sci Rep 9, |
| Case 12 | c.252_253dupGA,  p.Thr85Argfs*5 | 36 | female | immotile | NA | no | yes | NA | NA | NA | ODA+IDA | Tarkar (2013) Nat Genet 45, 995 |
| Case 13 | c.784-1037_894-2012del3549,  p.Trp262Glnfs*16 | 16 | male | immotile | NA | yes | yes | NA | NA | NA | ODA+IDA | Tarkar (2013) Nat Genet 45, 995 |
| Case 14 | c.(-256+1_-255-1)_(271+1_272-1) | NA | NA | NA | NA | NA | NA | NA | NA | NA | ODA+IDA | Blanchon (2020) J Med Genet 57, 237 |

Abbreviation: NA, not available.
